# Effect of body mass on physiological and time-trial performance responses to mixed-method cooling applied between exercise bouts in the heat

**DOI:** 10.3389/fphys.2026.1817856

**Published:** 2026-05-25

**Authors:** Takuma Yanaoka, Christopher J. Tyler, Hiroshi Hasegawa

**Affiliations:** 1 Graduate School of Humanities and Social Sciences, Hiroshima University, Hiroshima, Japan; 2 School of Life and Health Sciences, University of Roehampton, London, United Kingdom

**Keywords:** body composition, cardiovascular strain, cooling vest, half-time, ice slurry, perceptual strain, thermoregulation

## Abstract

This study investigated the effect of body mass (BM) on the efficacy of mixed-method cooling (ice slurry ingestion + cooling vest) applied between exercise bouts. Sixteen males completed two experimental trials in the heat (35°C, 50% relative humidity) in a randomized and counterbalanced order. In each trial, participants completed two 30-min cycling bouts (25-min cycling at 55% of maximal oxygen uptake followed by a 5-min time-trial) separated by a 15-min break. During the break, participants received mixed-method cooling (COOL: 5.0 g·kg^-1^ of ice slurry ingestion [-1.4 ± 0.2 °C] with a cooling vest) or no cooling (CON: 5.0 g·kg^-1^ of fluid ingestion [28.5 ± 2.7 °C]). Participants were categorized according to their BM values as Heavier (72.2 ± 6.9 kg, n=8) or Lighter (59.3 ± 4.1 kg, n=8). Compared with CON, COOL reduced rectal temperature during the break in the Heavier group (-0.32 °C, p=0.013, d=1.14) and at the end of the second bout in both groups (Heavier: -0.31 °C, p=0.005, d=0.90; Lighter: -0.26 °C, p=0.016, d=0.46). The difference in rectal temperature at the end of the break between trials correlated with BM (r=-0.506, p=0.046, n=16). In an additional linear mixed-model analysis of rectal temperature across all time points, BM was not a significant covariate (BM: p=0.650, BM×trial: p=0.120, BM×trial×time: p=0.275). Heart rate was lower in COOL during the break and second bout in the Heavier (p<0.05, d=0.46-0.88) and Lighter groups (p<0.05, d=0.25-0.72). COOL mitigated the decline in mean power output in the second time-trial compared to the first time-trial (p=0.027, d=0.47). However, there was no significant difference in the ergogenic effect between the groups. Mixed-method cooling during the 15-min break attenuated thermal and perceptual strain during subsequent 25-min exercise and improved cycling 5-min time-trial performance. Given that BM was not a significant covariate for rectal temperature, BM-related differences in the efficacy of mixed-method cooling should be interpreted cautiously.

## 1 Introduction

Exercise in the heat often results in increased physiological (e.g., elevated core body temperature [T_c_], skin temperature [ $\bar{\text{T}}$_sk_], and heart rate [HR]) and perceptual (e.g., increased rating of perceived exertion [RPE] and thermal sensation and decreased thermal comfort) strain (Alhadad et al., 2019; Périard et al., 2021), and so heat mitigation strategies are sought. As an acute heat-mitigation strategy, ice slurry ingestion can help alleviate physiological strain by decreasing T_c_, due to heat transfer between the fluid and internal tissues (Onitsuka et al., 2018). Moreover, ice slurry ingestion may decrease brain temperature due to inflow of cooled carotid blood and conductive cooling of the facial skin and brain (Onitsuka et al., 2018).

The magnitude of T_c_ reduction caused by ice slurry ingestion can be predicted from the heat sink created by the ice slurry and the mass of tissues cooled by it (Jay and Morris, 2018). Heavier individuals should have a greater internal heat sink because fixed-dose ice slurry ingestion protocols are typically prescribed relative to body mass (BM; 5.0–7.5 g·kg^-1^ BM) (Onitsuka et al., 2015; Takeshima et al., 2017; Yanaoka et al., 2022). In contrast, the mass of major internal organs, which likely constitute a substantial proportion of the tissues cooled by the ingested ice slurry, does not appear to increase proportionally with BM. For instance, in a study of normal organ weights, even the liver, the second-heaviest organ in the human body, increased by only approximately 1 kg despite an approximately 9 kg difference in BM (Bell et al., 2022). As a result, the corresponding rates of internal heat sink created by ice slurry per unit of the cooled tissue may be higher in heavier individuals than in lighter individuals. This may help explain why a typical ice-slurry ingestion protocol could produce a larger reduction in T_c_ in heavier individuals. Moreover, a large proportion of blood, which constitutes approximately two-thirds of visceral mass at rest, remains pooled in previously active skeletal muscle during post-exercise recovery, suggesting that the mass of tissues cooled by ice slurry may temporarily decrease during post-exercise recovery irrespective of BM differences (Kenny et al., 2006). Thus, a reduction in T_c_ by ingesting ice slurry in heavier individuals may amplify during a short break (10–20 min) between exercises as opposed to the pre-exercise period.

However, ingesting ice slurry may not necessarily result in a net cooling effect in subsequent exercise owing to compensatory reductions in sudomotor responses that can reduce evaporative heat loss (Jay and Morris, 2018). A previous study observed that, compared with individuals with a high body surface area (A_D_) to BM ratio (A_D_/BM), those with a low A_D_/BM are more dependent on sweat evaporation for heat loss during light and moderate exercise in the heat (Notley et al., 2016). Because heavier individuals generally have a lower A_D_/BM, any attenuation of sweating after ice slurry ingestion may reduce their evaporative heat loss during subsequent exercise to a greater extent. Therefore, although ice slurry ingestion may initially induce a larger reduction in T_c_ in heavier individuals, this advantage may be short-lived once exercise resumes. A potential way to avoid this would be to combine ice slurry ingestion with a cooling vest. The combined internal and external cooling approach could increase heat storage capacity by concurrently decreasing T_c_ and $\bar{\text{T}}$_sk_ (Thomas et al., 2019), resulting in a longer-lasting reduction in T_c_ and may improve exercise performance in the heat (Yanaoka et al., 2022). Moreover, cooling vests, which can cool the neck and torso, may have a greater effect on mitigating thermal perception to promote exercise performance in the heat (Chaen et al., 2019; Thomas et al., 2019). Although the relative cooling area provided by cooling vests may vary depending on the wearer’s body size, improvement in perceptual stress by wearing cooling vests appears to depend more on the cooling site (e.g., the neck) than the cooling area (Douzi et al., 2019). Nevertheless, it remains unknown whether anthropometric characteristics (e.g., BM) influence the effectiveness of practical acute cooling strategies such as mixed-method cooling using ice slurry ingestion and a cooling vest. Identifying low- and high-responders to such practical interventions would be valuable for individualized heat-mitigation strategies.

Previous studies examining cooling strategies during a short break between exercise bouts have primarily employed exercise performance tests (Onitsuka et al., 2015; Chaen et al., 2019) which provide effective performance assessment but makes accurate comparisons for physiological and perceptual responses difficult to make due to differences in exercise intensity and volume. For instance, Chaen et al. (2019) employed two 30-min cycling exercises separated by a 15-min break, with the exercise consisting of repetitions of a 5-s maximal sprint, 25-s active recovery, and 30-s passive recovery. This study reported a significant difference in mean power output during 5-s maximal sprints between the cool and non-cool conditions (Chaen et al., 2019). This result is likely to affect heat production in each condition. Pre-loaded time-trials are one way to enable accurate analysis for physiological and perceptual responses and recently one study reported that a 30-min preloaded time-trial (25-min constant-paced cycling followed by a 5-min performance test) can gain a clear, well-controlled understanding of the benefits of the cooling strategies with low within-subject coefficients of variation for rectal temperature (T_re_: 0.2%) and cycling time-trial (TT) performance (1.7%) (Yanaoka et al., 2022).

It seems prudent to suggest that the effectiveness of mixed-method cooling may be dependent on anthropometric differences between individuals; however, there is little empirical evidence to support this theoretical suggestion. Therefore, this study aimed to investigate the effect of body mass on the efficacy of mixed-method internal (ice slurry ingestion) and external (cooling vest) cooling administered in a short break between exercise bouts. We hypothesized that reductions in T_re_ during a short break would be greater and more sustained during subsequent endurance exercise in heavier males than in lighter males due to the greater internal heat sink and A_D_ cooled.

## 2 Methods

### 2.1 Participants

Sixteen physically active non-smoker males participated in this study. Participants were categorized according to their BM values (Heavier: top eight participants, Lighter: bottom eight participants). Participants were recruited from a university-based population, had a minimum of 6 years of sports participation, and exercised regularly (≥ 3 x 1.5 h sessions a week). Their sporting backgrounds varied from sprint/power-type (e.g., judo, track sprint events) to endurance-type sports (e.g., long-distance running events, triathlon). This study was approved by the Ethics in Human Research Committee of Hiroshima University (Approval number: 01-27), and all participants provided written informed consent to participate in this study.

### 2.2 Experimental design

To measure the physical characteristics and maximal oxygen uptake ( $\dot{\text{V}}$O_2max_), participants completed a preliminary test (visit 1). Stature was measured using a stadiometer (DC-270A-N, TANITA, Japan). BM, body fat percentage, lean body mass (LBM), and appendicular skeletal muscle mass (ASMM) were measured using multifrequency bioelectrical impedance analysis (InBody 470, InBody Japan, Japan). A_D_ was estimated using body height and BM measurements (Du Bois and Du Bois, 1989). An incremental ramp test was performed using a cycling ergometer (POWERMAX-V3 PRO, Konami Sports Life, Japan) to determine $\dot{\text{V}}$O_2max_ in a temperate environment (20 °C, 50% relative humidity). The test was initiated at 100 W with an increase of 20 W·min^-1^ until the participant could not maintain a pedaling cadence of at least 60 rpm. An automatic gas analysis system (AE 310S, Minato Medical Science, Japan) was used to measure $\dot{\text{V}}$O_2_. The criterion for achieving $\dot{\text{V}}$O_2max_ was a plateau in $\dot{\text{V}}$O_2_ despite an increase in exercise intensity, a respiratory exchange ratio of >1.15, or voluntary exhaustion. When the criteria for $\dot{\text{V}}$O_2max_ were not fully attained, $\dot{\text{V}}$O_2max_ was defined as the highest 20-s averaged value recorded during the final stage of the test. $\dot{\text{V}}$O_2max_ was matched between the groups because aerobic fitness strongly influences thermoregulatory responses during exercise in the heat (Yanovich et al., 2020). To achieve this, $\dot{\text{V}}$O_2max_ was assessed in all subjects prior to the experimental trials, and only individuals whose $\dot{\text{V}}$O_2max_ values could be matched between the heavier and lighter groups were included as participants before the first experimental protocol.

Participants then performed the same exercise protocol as the experimental trials for the familiarization trial (visit 2). For the experimental trials, participants completed two visits (visits 3 and 4) in a randomized and counterbalanced order. The familiarization and experimental visits were performed in the heat (35 °C, 50% relative humidity), separated by at least 5 days, and were performed at the same time of the day (± 1 h) for each participant to minimize any circadian rhythm-related variations. Participants were asked to record the meals and drinks consumed in the 24 h before the first experimental trial and repeat this in the 24 h before the second experimental trial. Participants refrained from strenuous training, alcohol, and caffeine for 24 h prior to each experimental trial and arrived at 3 h post-prandial, except for the consumption of water, for each experimental trial. This study was conducted during the winter in Japan to minimize possible heat acclimatization. All participants had not been exposed to temperatures exceeding 25 °C for at least eight weeks.

### 2.3 Experimental exercise

**Figure 1:**
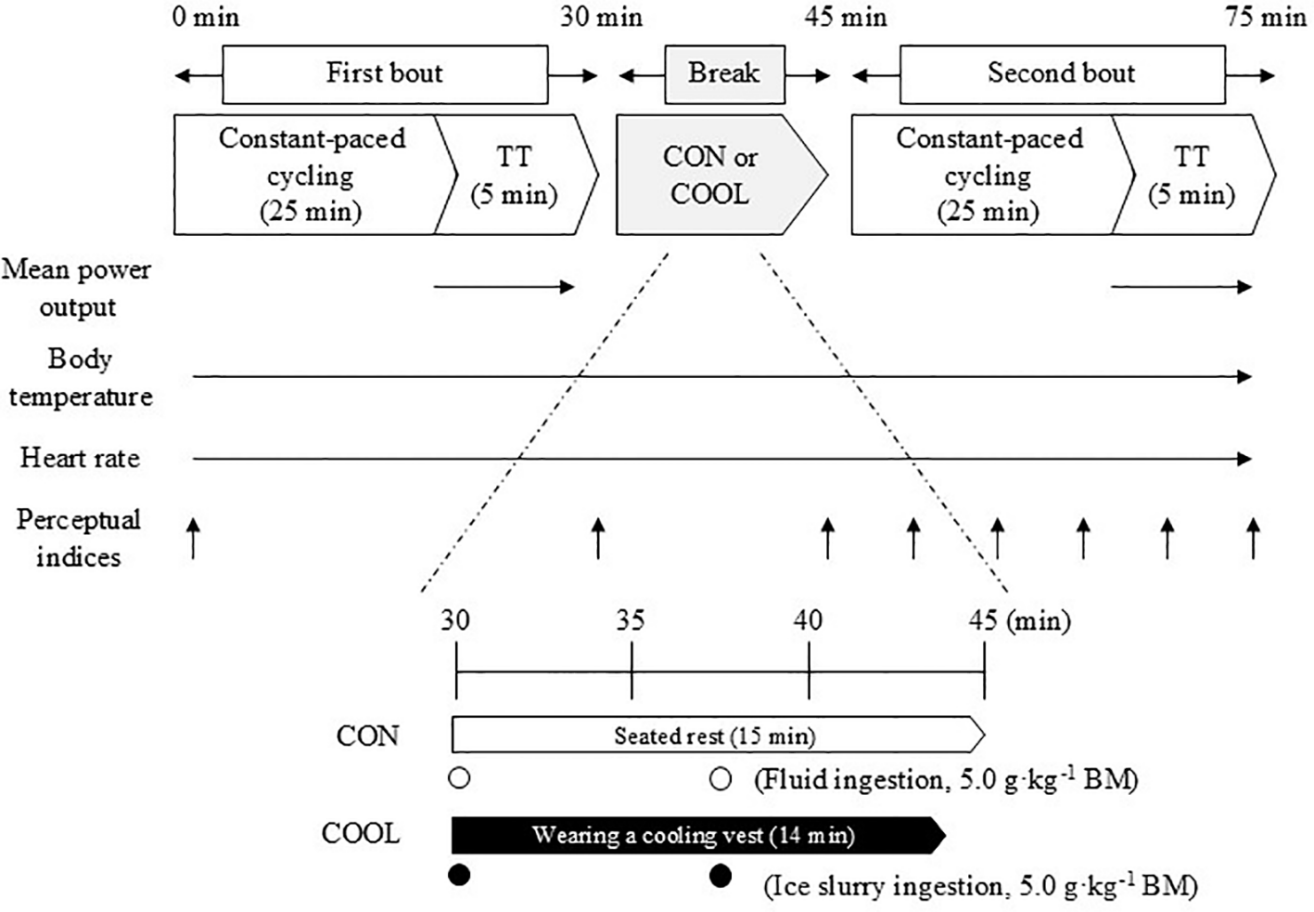
Schematic representation of the experimental exercise. TT, time-trial; BM, body mass.

All participants completed a standardized warm-up before two 30-min bouts of exercise separated by a 15-min break. Each 30-min bout consisted of 25-min constant-paced cycling at 55% of $\dot{\text{V}}$O_2max_ followed by a 5-min TT (**Figure 1**). Participants were instructed to complete as much work as possible during the 5-min TT. During the TT, external feedback was limited to the time taken by using a countdown clock. The participants consumed 2.5 g·kg^-1^ BM of water (29.0 ± 0.8 °C) at 15 min in each bout. The experimental exercise was identical to that in a previous study (Yanaoka et al., 2022).

### 2.4 Cooling intervention

During each 15-min break, participants sat in a hot environment (35 °C, 50% relative humidity) and received either mixed-method cooling (COOL; ingestion of 5.0 g·kg^-1^ BM of ice slurry [-1.4 ± 0.2 °C] while wearing a cooling vest) or no cooling (CON: ingestion of 5.0 g·kg^-1^ BM of fluid [28.5 ± 0.7 °C]). The cooling vest (Cooling Vest, Mizuno, Japan) was donned immediately after the first time-trial. The vest had eight pockets into which eight ice packs (approximately -5 °C) were inserted. The ice packs covered the neck (254 cm^2^), chest and abdominal region (240 cm^2^), back (494 cm^2^), and sides (240 cm^2^) for all participants. The vest was worn for 14 min because 1 min was required to put on and remove the vest. The ice slurry was prepared from a conventional sports drink (Pocari Sweat, Otsuka Pharmaceutical, Japan) containing 6.2 g of carbohydrates, 49 mg of sodium, and 20 mg of potassium per 100 mL. The total volume was split in half to enable even ingestion of the test beverages in both the trials and groups and participants were given 7.5 min to consume the bolus with the aid of a straw and spoon. The fluid in CON was the same sports drink as that in COOL. We utilized an ambient drink in CON to avoid overestimating the cooling effect of ice slurry ingestion compared with athletic settings.

### 2.5 Measurements

Upon arriving at the laboratory, urine specific gravity was measured to assess hydration using a refractometer (PAL-09S, Atago, Japan). If urine specific gravity was above 1.025 g·mL^-1^, participants consumed 540 mL of water to ensure they commenced each trial. After that, towel-dried nude BM was measured using a BM scale (HW-100KGV, A and D, Japan). A rectal thermistor (LT-ST08-21, Nikkiso-Therm, Japan) was self-inserted at a depth of 10 cm from the anal sphincter. A deep-tissue thermometer was attached at the right thigh to measure thigh deep-tissue temperature (T_deep-thigh_) employing the zero-heat-flow method (Yamakage and Namiki, 2003). Consistency between muscle temperatures measured using a needle thermocouple and the zero-heat-flow method has been reported (Togwa et al., 1976). Three skin thermistors (LT-ST08-12, Nikkiso-Therm, Japan) were attached at the chest, upper arm, and thigh. An insulation material pad (Temperature Insulation Pad, Nihon Kohden Corporation, Japan) was placed directly on the thermistor at the chest during the break to minimize heat flow between the skin and the ice pack. $\bar{\text{T}}$_sk_ was calculated as follows: $\bar{\text{T}}$_sk_ = (0.43 × chest) + (0.25 × upper arm) + (0.32 × thigh) (Roberts et al., 1977). A HR monitor (V800, Polar Electro, Finland) was attached. T_re_, T_deep-thigh_, $\bar{\text{T}}$_sk_, and HR were measured at 1-min intervals. Thermal sensation (-6 [very cold] to 6 [very hot]) and thermal comfort (-6 [very uncomfortable] to 6 [very comfortable]) were measured at 5-min intervals (Olesen and Brager, 2004). The RPE (6 [no exertion] to 20 [maximal exertion]) was measured at 5-min intervals during the exercise (Borg, 1982). The mean power output (MPO) during the TT was measured using a built-in computer in a cycling ergometer (POWERMAX-V3 PRO, Konami Sports Life, Japan). The change (Δ) in MPO between the first and second TTs was calculated. The change was expressed as both absolute power output and BM-adjusted power output (ΔMPO/BM). ΔMPO/BM was calculated as (MPO in the second TT − MPO in the first TT)/BM. Following the end of the second TT, towel-dried nude BM was measured using the same BM scale.

### 2.6 Sample size and statistical analyses

A sample size of eight participants was required in each group based on a power calculation (G*Power 3) (Faul et al., 2007), with an α, β, and effect size (partial η^2^) of 0.05, 0.20, and 0.184, respectively, calculated from T_re_ from a previous study that employed the same cooling method (Yanaoka et al., 2022).

Statistical analyses were performed using SPSS software (version 29.0, SPSS Japan Inc., Japan), and statistical significance was considered at p ≤ 0.05. All values are presented as mean ± standard deviation. The normality of the data was assessed using the Shapiro–Wilk test. An unpaired t-test was used to analyze the physical characteristics. Three-way (trial × time × group) analysis of variance was used to analyze T_re_, T_deep-thigh_, $\bar{\text{T}}$_sk_, and HR. Two-way (trial × group) analysis of variance was used to analyze ΔMPO, ΔMPO/BM, total sweat loss, and percentage of body fluid loss. Where significant main effects and interactions were found, *post hoc* pairwise comparisons with Bonferroni adjustment were performed. Perceptual indices were analyzed using Wilcoxon’s matched-pairs test. An association between BM and a difference in T_re_ at the end of the break between the trials in all participants was also assessed using Pearson’s product-moment test. In addition, to evaluate the influence of BM as a continuous variable, T_re_ was further analyzed using a linear mixed model including trial, time, BM, and their interaction terms as fixed effects. ΔMPO was also further analyzed using a repeated-measures analysis of covariance, with trial as the within-subject factor and BM as a covariate. Cohen’s d effect size was also presented, whereby >0.8, 0.5-0.8, 0.2–0.5, and <0.2 were categorized as large, moderate, small, and trivial effects, respectively (Cohen, 1988). The coefficient of variation for first TT, T_re_ at the end of the first bout, and BM among visits 1, 2, and 3 were used as a measure of reproducibility.

## 3 Results

### 3.1 Physical characteristics

**Table 1** summarizes the physical characteristics in each group. The Heavier group was taller and had higher BM, LBM, ASMM, body fat percentage, and A_D_ than the Lighter group (p<0.05). The Heavier group also had lower A_D_/BM, A_D_/LBM (p<0.05). In addition, the A_D_ covered by ice packs in the cooling vest was lower in the Heavier group than in the Lighter group (p<0.05). V̇O_2max_ was not significantly different between the groups (p=0.692).

**Table 1:** Physical characteristics in both groups.

	Heavier (n=8)		Lighter (n=8)		P value	Cohen’s d
Variables	Mean ±SD	Range	Mean ± SD	Range		
Age (yr)	21.0 ± 1.1	20.0 - 23.0	20.5 ± 1.6	19.0 - 23.0	0.475	0.37
Height (m)	1.75 ± 0.06	1.68 - 1.85	1.68 ± 0.02	1.65 - 1.71	0.009	1.50
BM (kg)	72.2 ± 6.9	65.6 - 83.0	59.3 ± 4.1	54.1 - 65.5	< 0.001	2.27
LBM (kg)	61.3 ± 5.7	56.3 - 70.5	52.4 ± 3.5	47.2 - 56.8	0.002	1.89
ASMM (kg)	35.0 ± 3.6	31.8 - 41.0	29.5 ± 2.0	26.5 - 32.2	0.002	1.92
Body fat (%)	15.0 ± 1.8	11.9 - 17.0	11.6 ± 1.7	8.9 - 13.2	0.001	1.98
A_D_ (m^2^)	1.87 ± 0.11	1.77 - 2.07	1.67 ± 0.06	1.58 - 1.76	< 0.001	2.28
A_D_/BM ratio (cm^2^·kg^-1^)	260 ± 12	238 - 272	282 ± 10	267 - 293	< 0.001	2.11
A_D_/LBM ratio (cm^2^·kg^-1^)	306 ± 14	287 – 324	320 ± 11	309 - 339	0.042	1.12
$\dot{\text{V}}$O_2max_ (mL·kg^-1^·min^-1^)	49.8 ± 7.7	39.1 - 60.5	51.6 ± 10.1	35.9 - 67.4	0.692	0.20

n = 16. BM, body mass; LBM, lean body mass, ASMM, appendicular skeletal muscle mass; A_D_, body surface area; $\dot{\text{V}}$O_2max_, maximal oxygen uptake.

### 3.2 Cycling time-trial performance

**Figure 2:**
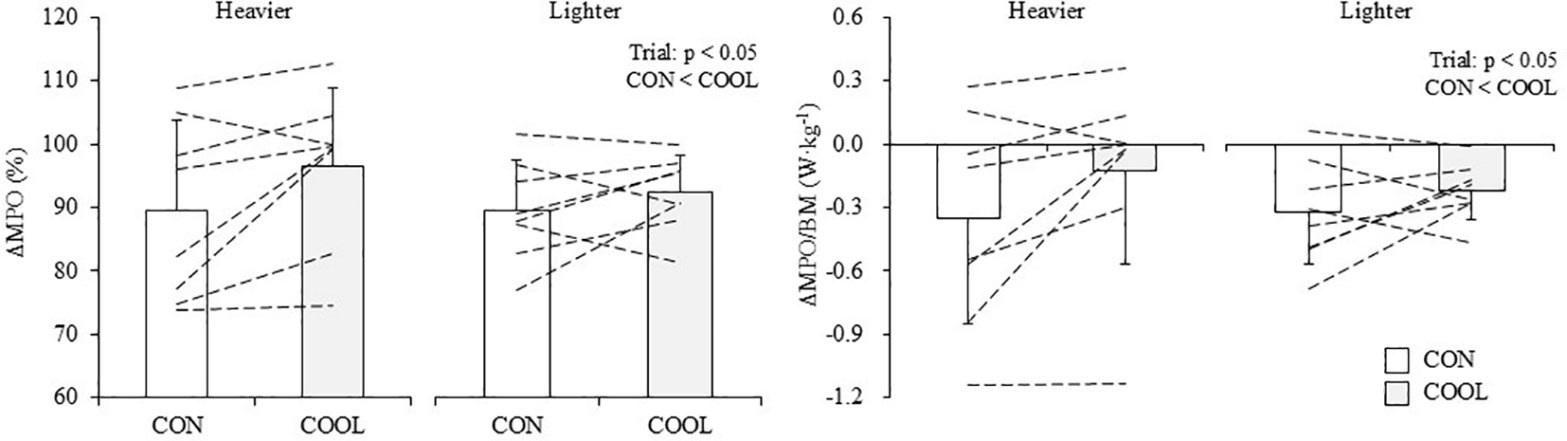
Changes in mean power output (ΔMPO) and BM-adjusted mean power output (ΔMPO/BM) during the time-trial (TT) between bouts in Heavier and Lighter groups. Bars show mean ± standard deviation (Heavier: n = 8, Lighter: n = 8). Dotted lines represent individual responses.

**Figure 2** shows ΔMPO and ΔMPO/BM during the TT in both groups. There was no trial × group interaction for ΔMPO (p=0.303). ΔMPO was significantly higher in COOL than in CON (p=0.027, d=0.47). In an additional repeated-measures analysis of covariance treating BM as a continuous covariate, there were no significant effects of BM (p=0.815) or trial × BM interaction (p=0.440). The within-subject coefficient of variation for first TT between trials was 2.5 ± 2.8%.

There was no trial × group interaction for ΔMPO/BM (p=0.371). ΔMPO/BM was significantly higher in COOL than in CON (p=0.029, d=0.46). In an additional repeated-measures analysis of covariance treating BM as a continuous covariate, there were no significant effects of BM (p=0.692) or trial × BM interaction (p=0.989).

### 3.3 Body temperatures

**Figure 3:**
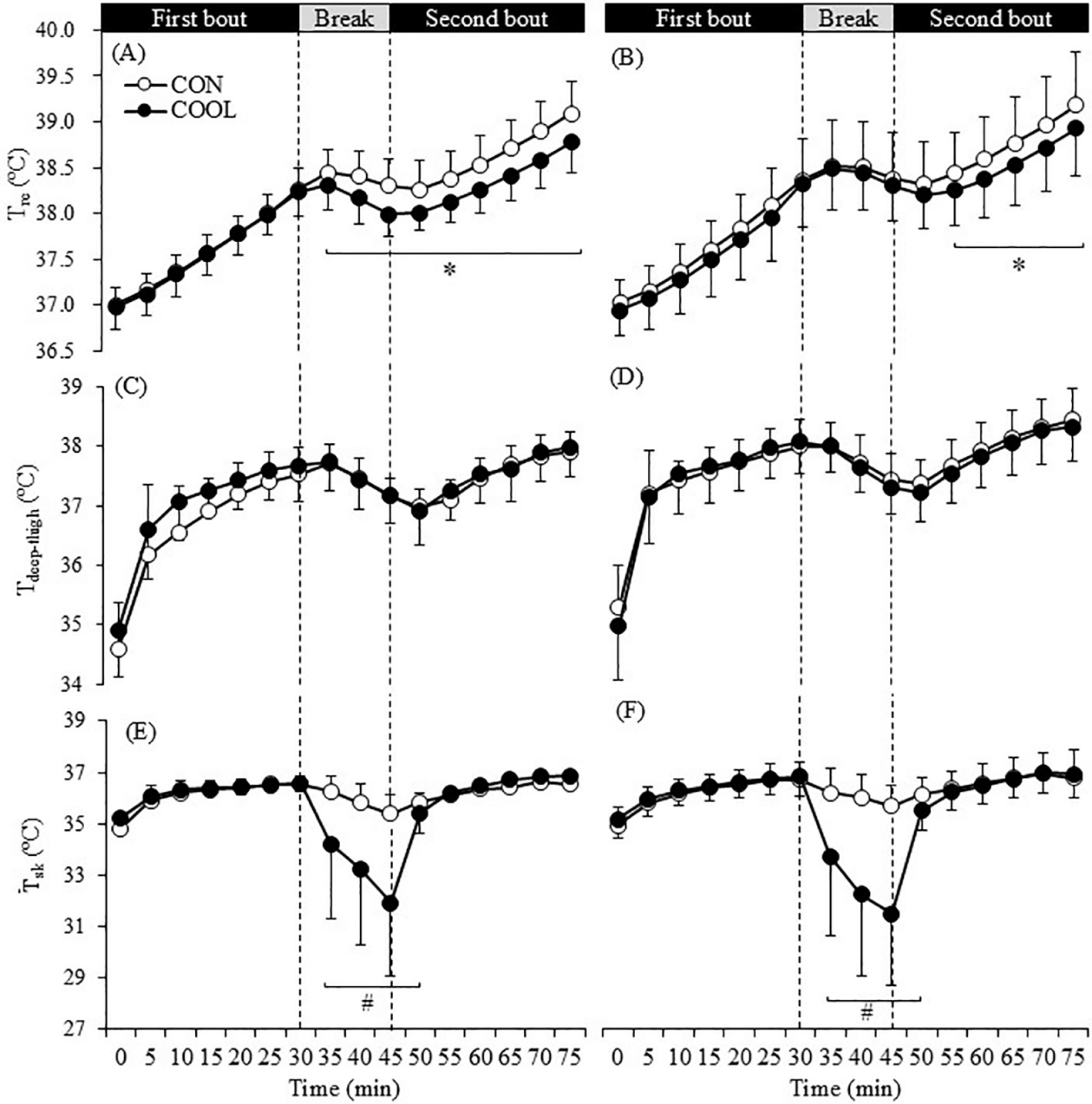
Rectal (T_re_), thigh deep-tissue (T_deep-thigh_), and mean skin ( $\bar{\text{T}}$_sk_) temperatures in Heavier **(A, C, E)** and Lighter **(B, D, F)** groups. Values are shown as mean ± standard deviation (Heavier: n = 8, Lighter: n = 8). *Significant difference between trials in trial×time×group interaction (p<0.05). ^#^Significant difference between trials in trial×time interaction (p<0.05).

**Figure 4:**
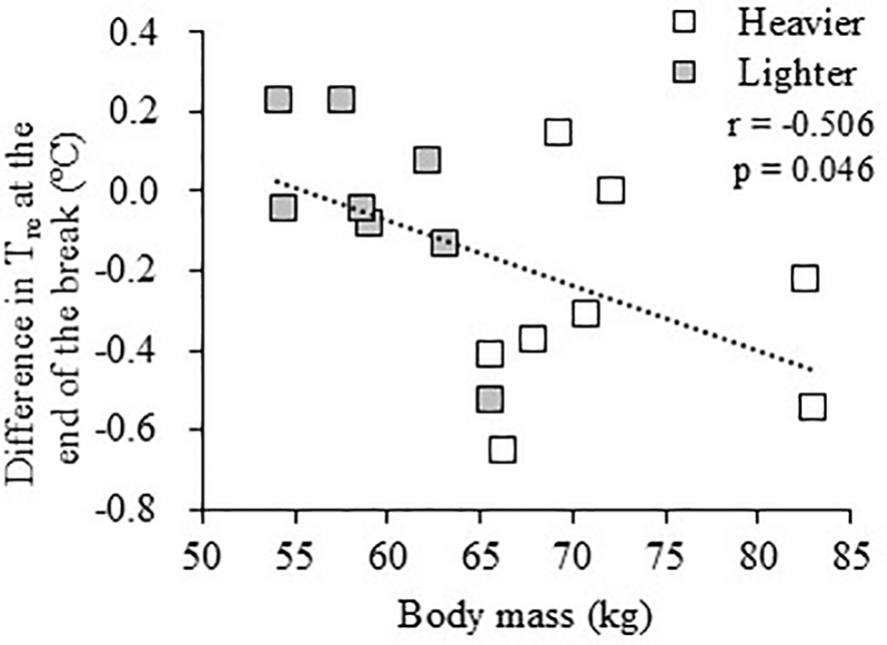
Correlations between body mass and differences in rectal temperature (T_re_) at the end of the break between the trials in all participants (n = 16).

There was a trial × time × group interaction for T_re_ (p<0.001, **Figures 3A, B**). In the Heavier group, T_re_ during the break and second exercise bout was significantly lower in COOL than in CON (35 min: p=0.043, d=0.51, 40 min: p=0.002, d=0.87, 45 min: p=0.001, d=1.14, 50 min: p=0.005, d=0.89, 55 min: p=0.003, d=0.89, 60 min: p=0.002, d=0.94, 65 min: p=0.002, d=0.97, 70 min: p=0.002, d=0.99, 75 min: p=0.005, d=0.90). In the Lighter group, T_re_ during the break and second exercise bout was significantly lower in COOL than in CON (55 min: p=0.021, d=0.40, 60 min: p=0.008, d=0.48, 65 min: p=0.008, d=0.49, 70 min: p=0.010, d=0.47, 75 min: p=0.016, d=0.46). There were no significant differences between the groups at all time points in both trials (p > 0.05). In COOL, there was a small effect size between the groups at 45 min (-0.32 °C for the Heavier group, p=0.070, d=0.32). The difference in T_re_ at the end of the break between the trials (i.e., substantial T_re_ lowering effect induced by the mixed-method cooling) correlated with BM (r=-0.506, p=0.046, n=16, **Figure 4**). In an additional linear mixed-model analysis treating BM as a continuous covariate across all time points, there was a significant BM × time interaction (p=0.030). There were no significant effects of BM (p=0.650), BM × trial (p=0.120), or BM × trial × time interaction (p=0.275). The within-subject coefficient of variation for T_re_ at the end of the first bout was 0.2 ± 0.1%.

There was no trial × time × group interaction for T_deep-thigh_ (p=0.211). There was also no main effect of trial (p=0.607) (**Figure 3**). There was a trial × time interaction (p=0.002) for T_deep-thigh_. *Post-hoc* tests revealed no significant differences between trials for T_deep-thigh_ throughout the experimental trial (p>0.05).

There was no trial × time × group interaction for $\bar{\text{T}}$_sk_ (p=0.994), but there was a trial × time interaction for $\bar{\text{T}}$_sk_ (p<0.001, **Figure 3**). $\bar{\text{T}}$_sk_ was significantly lower in COOL than in CON at 35 min (p=0.008, d=0.94), 40 min (p<0.001, d=1.29), 45 min (p<0.001, d=1.81), and 50 min (p=0.002, d=0.69).

### 3.4 Heart rate and body fluid balance

**Figure 5:**
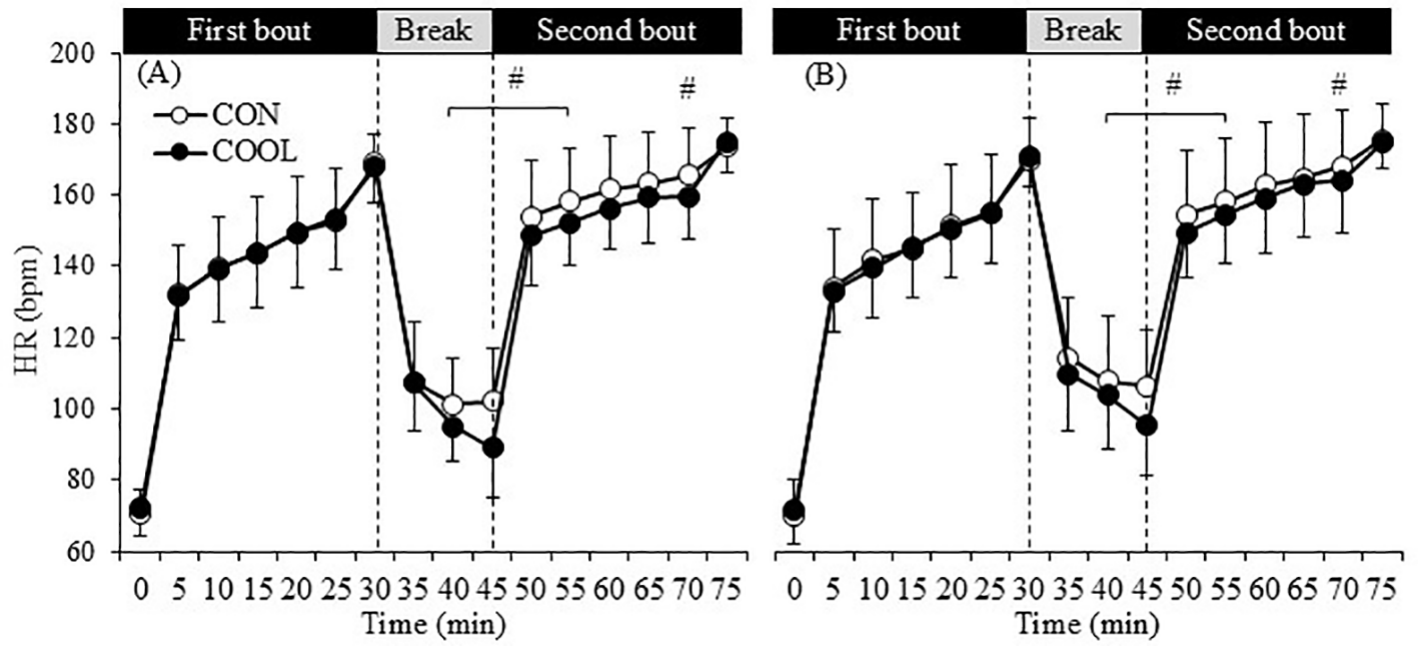
Heart rate (HR) response in Heavier **(A)** and Lighter **(B)** groups. # Significant difference between trials in trial × time interaction (p<0.05).

There was no trial × time × group interaction for HR (p=0.959). There was a trial × time interaction (p<0.001, **Figure 5**). HR was significantly lower in COOL than in CON at 40 min (p=0.029, d=0.31), 45 min (p<0.001, d=0.79), 50 min (p=0.037, d=0.31), 55 min (p=0.026, d=0.30), and 70 min (p=0.014, d=0.32).

There was no trial × group interaction for total sweat loss (p=0.808). There was also no main effect of trial (p=0.468). Total sweat loss was 2.11 ± 0.68 kg in CON and 2.07 ± 0.60 kg in COOL in the Heavier group, and 1.79 ± 0.51 kg in CON and 1.71 ± 0.60 kg in COOL in the Lighter group. There was no trial × group interaction for percentage of body fluid loss (p=0.777). There was also no main effect of trial (p=0.397). Percentage of body fluid loss was 1.94 ± 0.99% in CON and 1.88 ± 0.84% in COOL in the Heavier group, and 2.00 ± 0.91% in CON and 1.88 ± 1.10% in COOL in the Lighter group. The within-subject coefficient of variation for BM among visits 1, 3, and 4 was 0.7 ± 0.6%.

### 3.5 Perceptual responses

**Figure 6:**
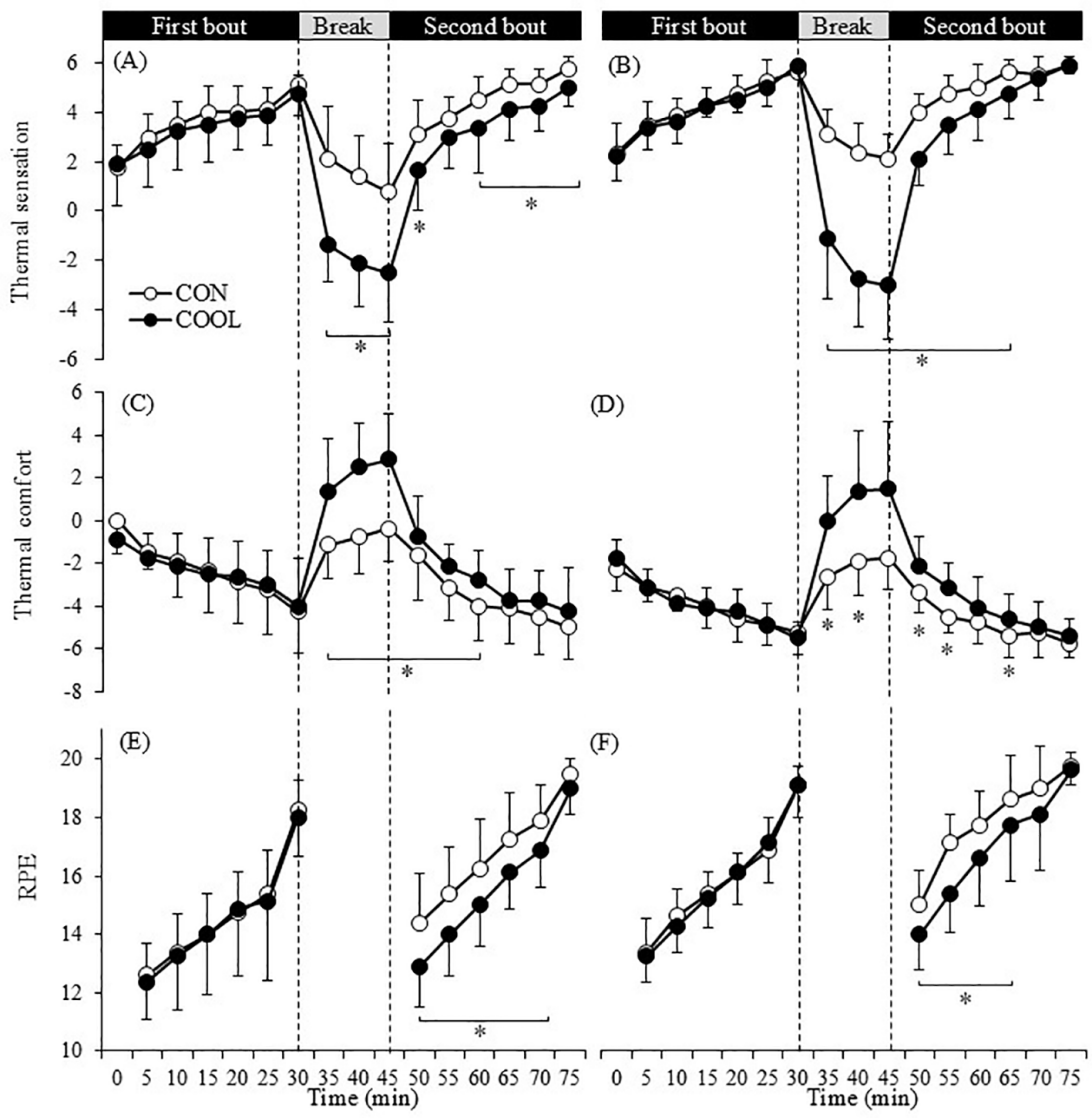
Thermal sensation, thermal comfort, and rating of perceived exertion (RPE) in Heavier **(A, C, E)** and Lighter **(B, D, F)** groups. *Significant difference between trials.

**Figure 6** shows perceptual responses throughout the experimental exercise. All indices at the initial phase of the second bouts (i.e., 50 min) were significantly improved by COOL compared to CON in both groups (p<0.05, **Figure 6**). Thermal sensation and RPE in the Heavier group remained improved until the commencement of the second TT (i.e., 70 min, p<0.05).

## 4 Discussion

To the best of our knowledge, this is the first study to examine whether the efficacy of mixed-method cooling during a short break between exercise bouts differs between heavier and lighter males. We employed a pre-loaded cycling TT (two 25-min constant-pace exercise followed by a 5-min TT) for accurate comparisons of cooling responses between the trials. In partial accordance with our hypotheses, during the break, higher BM was associated with a greater reduction in T_re_, and the cooling-induced reductions in T_re_ were larger in the Heavier group than in the Lighter group. In the second exercise bout, T_re_ was significantly lower in COOL than in CON in both groups. Moreover, cycling TT performance in the second exercise bout was significantly higher in COOL than in CON in both groups. However, there was no significant difference in the ergogenic effect between the groups.

A novel finding of this study was that, during the break between exercise bouts, mixed-method cooling lowered T_re_ in the Heavier group but not in the Lighter group (**Figure 3A**). Previous studies suggest that the primary effects of cooling vests during short breaks between exercise bouts are reductions in $\bar{\text{T}}$_sk_ and perceptual strain, whereas their direct contribution to core cooling is likely limited because the A_D_ covered by the vest is relatively small (Chaen et al., 2019; Thomas et al., 2019); thus, the reduction in T_re_ observed in the present study was driven mainly by ice slurry ingestion. The heat sink created by ice slurry ingestion is because of the thermodynamic characteristics of water and changing physical states; therefore, the magnitude of T_re_ reduction within the same participant is associated with the dose of ice slurry ingested (Ross et al., 2011). The present study employed a fixed dose per BM of ice slurry ingestion and, thus, the absolute heat sink created by ice slurry was higher in the Heavier group than in the Lighter group due to the difference in BM between groups. In contrast, most of the differences in BM (12.9 kg) between groups were due to differences in fat mass (4.0 kg) and ASMM (5.5 kg), suggesting that the between-group difference in the total amount of tissue expected to be cooled by ice slurry may have been smaller than the difference in BM itself. In addition, the total amount of tissue expected to be cooled by ice slurry may temporarily decrease during post-exercise recovery because a large proportion of blood remains pooled in previously active skeletal muscle (Kenny et al., 2006). Therefore, the difference in corresponding rates of internal heat sink induced by ice slurry per unit of cooled tissue between groups is likely to be even larger during post-exercise recovery. This greater relative internal cooling effect may help explain the greater reduction in T_re_ observed in the Heavier group during the break. Because the association between BM and the reduction in T_re_ was observed during the break, it was unlikely to be mainly attributable to factors influencing thermoregulatory responses during the subsequent exercise after ice slurry ingestion (e.g., tolerance of a greater workload and the resulting increase in total heat production, reductions in sweating, humidity, or exercise mode) (Convit et al., 2024).

Moreover, the present study showed that a reduction in T_re_ observed at the end of the break in COOL remained during the second bout in the Heavier group (i.e., 45–75 min, **Figure 3A**). This differs from a previous suggestion that ice slurry ingestion does not necessarily improve net body cooling during the subsequent exercise because reductions in sweating can offset the additional internal heat loss (Jay and Morris, 2018). This may be attributable to the combined use of a cooling vest and ice slurry ingestion. In a previous study using the same experimental protocol as the present study, ice slurry ingestion with a cooling vest was shown to lower T_re_ and $\bar{\text{T}}$_sk_ and to create a larger heat sink (Yanaoka et al., 2022). This may help explain why T_re_ in COOL remained lower than in CON from 45 to 75 min in the Heavier group. Additionally, the previous study suggested no difference in total sweat loss during the subsequent exercise bout when using a mixed-method cooling strategy between exercise bouts (Yanaoka et al., 2022). Another study also reported no difference in sweat rates during a running TT when using mixed pre-cooling strategies in females (Convit et al., 2024). These findings are consistent with the present result, which found no difference in total sweat loss. Taken together, the sustained T_re_ reduction in the Heavier group may reflect the cooling power of the combined use of a cooling vest and ice slurry ingestion, without negative impact on estimated total sweat loss under the present humidity conditions.

In the Lighter group, no reduction in T_re_ was observed during the break in COOL compared with CON, but T_re_ in COOL was lower than in CON during the second exercise bout (i.e., 55–75 min, **Figure 3B**). This afterdrop in T_re_ is similar to that observed in previous studies where participants with comparable BM consumed 7.5 g·kg^-1^ BM of ice slurry after warm-up (Takeshima et al., 2017) and during a break between exercise bouts (Onitsuka et al., 2015). This may be due to the thermoregulatory response and physical characteristics of the Lighter group. A previous study showed that individuals with a higher A_D_/BM were predisposed to dissipate heat via cutaneous vasodilation (Notley et al., 2016), suggesting that reductions in $\bar{\text{T}}$_sk_ by external cooling may be more effective for thermoregulation in the Lighter group. Moreover, the relative A_D_ cooled by the vest was larger in the Lighter group than in the Heavier group (**Table 1**) since the same cooling vest was employed between groups. These factors may have contributed to the delayed reduction in T_re_ observed during the second exercise bout in the Lighter group. However, we did not include isolated ice slurry or cooling-vest trials and the independent and combined contributions of each cooling strategy to the observed responses cannot be concluded. Moreover, the lower LBM in the Lighter group may have been associated with lower absolute metabolic heat production. Due to these limitations, additional research on this topic is warranted.

In both groups, ΔMPO and ΔMPO/BM during the second TT were significantly higher in COOL than in CON (**Figure 2**). This ergogenic effect in the present study aligns with a previous study suggesting that the performance benefit of pre-cooling is greater in self-paced exercise of short-to-medium duration (< 40 min) (van de Kerkhof et al., 2024). One of the mechanisms that may have contributed to the improvement in TT performance was the reduction in thermal and cardiovascular strain, as evidenced by lower T_re_ and HR at 70 min in COOL than in CON. In addition, given that reductions in T_re_ (Heavier: -0.31 °C, Lighter: -0.25 °C) and HR (Heavier: -5.9 bpm, Lighter: -4.1 bpm) at 70 min were relatively small, improvements in thermal sensation, thermal comfort, and RPE may also have contributed to ΔMPO and ΔMPO/BM during the second TT. Both internal (Onitsuka et al., 2018) and external (Chaen et al., 2019) cooling can reduce perceived strain. Moreover, ice slurry ingestion with a cooling vest has been shown to reduce perceived strain for at least 30 min during subsequent exercise when applied to females prior to exercise (Convit et al., 2025) and to males between exercise bouts (Yanaoka et al., 2022). The improved thermal sensation, thermal comfort, and RPE in the present study are in line with those studies. A notable finding in the present study is that reduced perceptual strain in COOL remained at the last part of the second bout in the Heavier group (**Figure 6**). Additionally, although not statistically significant, the magnitude of the decrease in T_re_ at 70 min was greater in the Heavier group (d=0.99) than in the Lighter group (d=0.47). However, there was no trial × group interaction for ΔMPO and ΔMPO/BM. These findings suggest that group differences in improvements in thermal and perceptual strain with mixed-cooling may be too small to elicit a meaningful ergogenic effect for 5-min cycling TT.

Several limitations of the present study should be acknowledged. First, the sample size was small (n=16), and participants were categorized based on BM. This grouping approach was a simplified way of examining inter-individual differences and may have amplified apparent group differences. In addition, the observed correlation between BM and the reduction in T_re_ during the break was moderate (r=0.506) and should therefore be interpreted with caution. Second, the groups differed not only in BM but also in other anthropometric characteristics, including LBM, A_D_, and body fat percentage, making it difficult to isolate the independent influence of BM alone. Although we performed additional analyses treating BM as a continuous variable, the present findings should still be interpreted cautiously in view of the small sample size and the interrelated nature of these body-composition variables. Third, the present protocol consisted of two 30-min exercise bouts separated by a 15-min break, and therefore the findings may be specific to this exercise format. The present findings may be more applicable to sports or exercise settings involving repeated bouts separated by a relatively long recovery period, rather than continuous endurance events. Fourth, because isolated ice slurry and cooling vest trials were not included, the independent and combined contributions of each cooling strategy to the observed responses cannot be separated. Finally, body composition was assessed using multifrequency bioelectrical impedance analysis rather than dual-energy X-ray absorptiometry. Although this method has been reported to correlate well with dual-energy X-ray absorptiometry (Alves et al., 2014; Lee et al., 2018), potential measurement error should be acknowledged.

In conclusion, mixed-method cooling during the 15-min break attenuated thermal and perceptual strain during subsequent exercise and improved performance in the second cycling TT. During the break, a correlation was observed between BM and the reduction in T_re_, and the reduction in T_re_ was larger in the Heavier group. However, no significant effect of BM was observed when BM was analyzed as a continuous covariate in a linear mixed model. Therefore, BM-related differences in the efficacy of mixed-method cooling should be interpreted cautiously.

## Data Availability

The raw data supporting the conclusions of this article will be made available by the authors, without undue reservation.
